# Spatially distributed and interconnected porous architectures for dental implants

**DOI:** 10.1186/s40729-025-00618-6

**Published:** 2025-04-07

**Authors:** Rana Dabaja, W. Benton Swanson, Sun-Yung Bak, Gustavo Mendonca, Yuji Mishina, Mihaela Banu

**Affiliations:** 1 Department of Mechanical Engineering, University of Michigan, 2350 Hayward St, Ann Arbor, MI 48109 USA; 2 Department of Biologic and Materials Sciences & Prosthodontics, School of Dentistry, University of Michigan, 1011 N University Ave, Ann Arbor, MI 48109 USA; 3 Department of General Practice, Virginia Commonwealth University School of Dentistry, Richmond, VA USA; 4 Present Address: Harvard University School of Dental Medicine, 188 Longwood Ave, Boston, MA 02115 USA

**Keywords:** Selective laser melting, Dental implant, Porous architecture, Cell proliferation, Permeability, Triply periodic minimal surface, Stochastic

## Abstract

**Purpose:**

Patients with pre-existing medical conditions that impair bone integrity face challenges in dental implant success due to compromised osseointegration. This study evaluates three titanium interconnected porous architectures: the TPMS solid gyroid, TPMS sheet gyroid, and Voronoi stochastic lattice. We aim to assess manufacturability, design controllability, and cellular interactions to identify an optimal architecture that enhances cellular behavior with the potential to strengthen bone-to-implant contact.

**Methods:**

Three porous architectures were designed and compared: the two variants of the uniform, periodic triply periodic minimal surface (TPMS) gyroid, and the random, non-uniform Voronoi stochastic lattice. The porous constructs were fabricated using selective laser melting (SLM) and evaluated using microcomputed tomography (microCT) for porosity, manufacturability, and permeability. In vitro experiments used primary bone marrow stromal cells (BMSCs) isolated from 8-week-old wild type C57BL6/J mice. These cells were seeded onto the SLM-fabricated porous architectures and evaluated for adhesion using scanning electron microscopy (SEM) and RNA extraction. Cell trajectory was profiled using fluorescent confocal microscopy.

**Results:**

Selective laser melting (SLM) successfully fabricated all three porous architectures, with the TPMS solid gyroid exhibiting the highest manufacturing resolution, controllability, and the most uniform pore distribution. Computational fluid dynamics (CFD) analysis showed that its permeability outperformed both the TPMS sheet gyroid and stochastic Voronoi architectures. In vitro cell culturing demonstrated superior cell behavior in the TPMS solid gyroid scaffold. RNA quantification after 72 h of culture showed that cells are most adherent to the TPMS solid gyroid, demonstrating a 4-fold increase in RNA quantity compared to the fully dense (control). Additionally, cell trajectory analysis indicated enhanced cell infiltration and cellularization within the pore channels for the TPMS solid gyroid architecture.

**Conclusion:**

This research demonstrates that inducing an interconnected porous architecture into a titanium construct enhances cellular behavior compared to a traditional dense implant. The TPMS solid gyroid architecture showed superior manufacturability, making it a promising solution to improve dental implant success in patients with compromised bone integrity.

## Background

The dental implant market is projected to grow from USD 4.42 billion in 2023 to USD 6.95 billion by 2030, reflecting a rising demand for dental restoration procedures [1]. Despite this growth, implant failure rates remain as high as 10%, often attributed to oral complications arising from pre-existing conditions such as uncontrolled diabetes and periodontal disease [2, 3]. These conditions impair osseointegration, negatively affecting wound healing, bone regeneration, and bone mass density [3–9]. A study on medically compromised patients with low bone mass density revealed dental implant success rates as low as 77.5% [2].

Osseointegration, the direct connection between living bone and the surface of an implant, is crucial for long-term implant success [3, 10, 11]. Primary stability, occurring upon implant placement, is influenced by bone quality and the bone-implant interface [12–14]. After implant placement, the bone remodels and grows around the treads to promote secondary stability and biological osseointegration with the host bone tissue [13]. Enhancing secondary stability, through improved biological interactions, will ensure long-term success for the dental implant [15]. Improved secondary stability can be achieved through methods such as implant design, material, and surface modifications.

The titanium alloy Ti6Al4V Extra Low Interstitial (ELI) Grade 23 is an industry-standard material for dental implants, favored for its superior biocompatibility, osseointegration capacity, and mechanical properties [16]. Compared to commercially pure titanium, Ti6Al4V ELI Grade 23 offers higher strength, corrosion resistance, and ductility, making it ideal for high-stress medical implant applications. It also outperforms Ti6Al4V Grade 5 by having lower levels of interstitial elements (iron, carbon, nitrogen, and oxygen), which enhances corrosion resistance, fracture toughness, and ductility, making it well-suited for long-term implant applications [16–18]. However, traditional solid Ti6Al4V implant designs face limitations in promoting cell proliferation and adhesion, particularly in patients with large bone defects or compromised bone quality.

Current surface modification strategies such as nanoroughening by sandblasting, aim to increase surface roughness and have shown to enhance osteoblast adhesion, bone remodeling, and duration of healing by increasing surface area [11, 19]. These surface modifications only promote bone apposition, where new bone forms on the implant surface rather than integrating within it. The surface level interaction is often insufficient in compromised bone as it does not address long-term biological fixation or prevent marginal bone loss over time [20]. While roughened surfaces improve biological interactions, they facilitate bacterial adhesion and peri-implantitis, especially in periodontally diseased patients [21, 22].

These challenges highlight the need for advanced dental implant designs that address biological and mechanical performance. To overcome these limitations, embedded porous architectures into a titanium implant have emerged as a promising alternative, offering a structure that not only enhances bone apposition but integrates with the surrounding bone tissue for guided bone regeneration in a compromised bone environment. Unlike traditional surface modifications, interconnected porous architectures provide a scaffold structure that acts as an extracellular matrix, offering a framework for cell migration, vascularization, and mechanical interlocking with newly formed bone tissue to enhance bone-to-implant contact [23].

Suitable pore architectures, defined by parameters such as size, porosity, geometry, and interconnectivity, are fundamental for enhancing biological interactions. Studies suggest that pore sizes ranging from 200 to 425 μm, with a minimum of 125 μm for vascularization, are optimal for bone growth [24–26]. Among porous designs, lattice structures have gained prominence for biomedical applications driven by their structural characteristics and adjustable mechanical properties [27]. More precisely, the two lattice configurations most favorable for biomedical applications are the triply periodic minimal surface (TPMS) gyroid and stochastic structure using the Voronoi tessellation method. TPMS lattices are continuous and periodic in three independent perpendicular directions and highly interconnected, while Voronoi stochastic lattices mimic the non-uniformity and randomness of bone [28–31]. TPMS structures feature continuous, highly interconnected pores and mimic natural biological architectures found in exoskeletons and membranes, and specifically, the gyroid TPMS structure offers superior surface area, permeability, and mechanical strength [31–33].

Currently, conventional manufacturing methods, such as subtractive manufacturing, limit the development of porous designs necessary for implants. The advent of additive manufacturing, specifically selective laser melting (SLM), has enabled the precise fabrication of complex porous designs for titanium implants. SLM is particularly well-suited for producing intricate microporous structures due to its superior resolution and is the most common for implant applications for Ti6Al4V and fabricating metallic implants [34–36]. Its notable technique in additive manufacturing utilizes a laser beam to selectively melt metal powders layer by layer to create a three-dimensional product. Previous studies have demonstrated the successful fabrication of porous scaffolds using SLM. For instance, Wang et al., fabricated gyroid scaffolds with pore sizes between 720 and 1763 μm, finding that smaller pores supported greater cell adhesion and proliferation due to increased surface area [37]. Porous materials, while increasing surface area for enhanced cell attachment, compromise mechanical strength, impacting their ability to withstand physiological loading [38, 39]. Hybrid designs incorporating a dense core have been developed to address the mechanical limitations of highly porous structures, balancing biological performance with structural integrity [40]. A study done by Xiong et al., conducted compression and fatigue tests of an SLM-fabricated TPMS gyroid structure with a pore size of 400 μm, demonstrating that the insertion of a dense core into the porous structure is an effective way to strengthen the mechanical properties while maintaining favorable porosity for bone ingrowth and cell infiltration [41]. Despite these advancements, direct comparisons between TPMS gyroid and Voronoi stochastic architectures remain limited, particularly regarding biological performance, permeability, and manufacturability. This study aims to fill this gap by evaluating the biological interactions and manufacturability of these three bioinspired porous configurations to drive an optimal design choice. We hypothesize that the TPMS solid gyroid, TPMS sheet gyroid, and the Voronoi stochastic architectures will demonstrate more favorable biological interactions compared to a traditional solid dental implant, with the TPMS solid gyroid structure offering superior cellular response and ease of fabrication. By embedding a scaffold design in a dental implant to mimic the function of healthy bone, this research seeks to offer a transformative solution for patients with pre-existing conditions negatively affecting bone quality and currently limiting implant eligibility.

## Methods

### Design of the porous architectures

Three lattice methods were carefully selected to achieve a structure that closely replicates natural bone. These methods include the Voronoi stochastic structure, solid network gyroid TPMS, and sheet network gyroid TPMS, all designed using nTop, Release 3.25.3 (nTop, Inc, New York, USA).

TPMS structures can be categorized into two distinct groups, solid network, and sheet network structures. These categories are characterized by their respective mathematical models described in Eqs. 1 and 2. The design of the pores structures depends on user-defined values for the geometrical parameters L, the unit cell size applied in the x-y-z cartesian coordinates direction, and c the isovalue constant that controls the wall thickness [42]. Equation 1 models the solid network gyroid where the thickness is exclusively applied in one direction, either $\:\phi\:\left(x,y,z\right)<c\:$or undefined$\:\phi\:\left(x,y,z\right)>c$c]]> and Eq. 2 models the sheet-network Gyroid where c is simultaneously applied in both directions [42, 43].

1 $$\eqalign{\:\phi {\:_G} \equiv \: & \cr si & n\:\left( {\frac{{2\pi \:}}{L}x} \right){\text{cos}}\left( {\frac{{2\pi \:}}{L}y} \right) & \cr &+ sin\:\left( {\frac{{2\pi \:}}{L}y} \right){\text{cos}}\left( {\frac{{2\pi \:}}{L}z} \right) \cr & + sin\:\left( {\frac{{2\pi \:}}{L}z} \right){\text{cos}}\left( {\frac{{2\pi \:}}{L}x} \right) = c \cr}$$

2 $$\eqalign{\phi {\:_G} \equiv \, \cr & \:sin\:\left( {\frac{{2\pi \:}}{L}x} \right){\text{cos}}\left( {\frac{{2\pi \:}}{L}y} \right) & \cr & + sin\:\left( {\frac{{2\pi \:}}{L}y} \right){\text{cos}}\left( {\frac{{2\pi \:}}{L}z} \right) & \cr & + \:sin\:\left( {\frac{{2\pi \:}}{L}z} \right){\text{cos}}\left( {\frac{{2\pi \:}}{L}x} \right) = \pm \:c \cr} $$

**Fig. 1:**
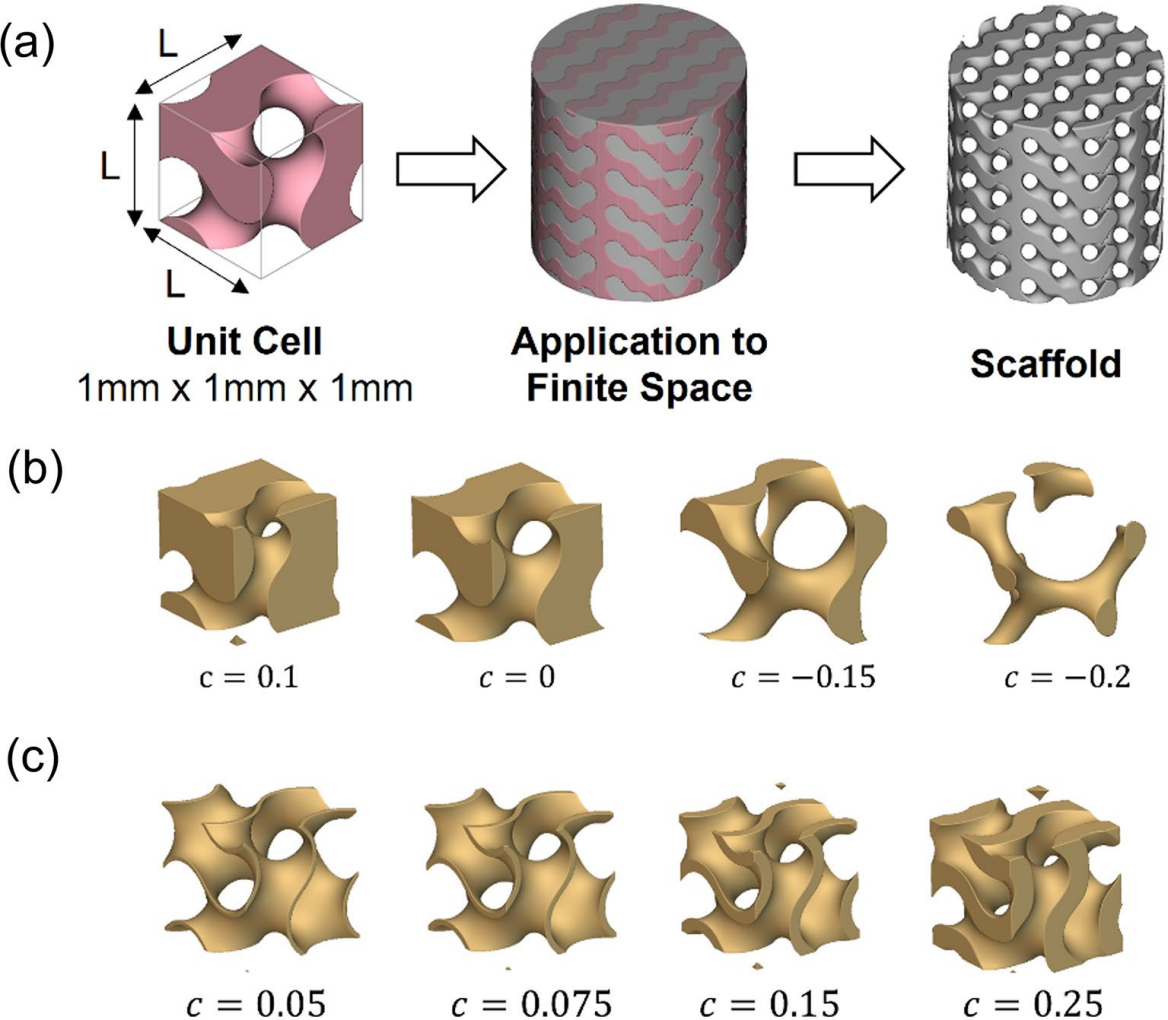
(**a**) Design process for a TPMS gyroid structure with a defined unit cell geometry applied to a finite space to model a scaffold. The two types of TPMS gyroid structures: (**b**) The solid network TPMS gyroid unit cell thickens the c constant in one direction; (**c**) The sheet network thickens the c constant in the positive and negative direction to create more surface area

Increasing L increases the size and periodicity of the pores of the unit cell in the x-y-z direction, and increasing c increases the wall thickness, which decreases pore size. The TPMS unit cell created using the equations is then assigned to a specific finite shape to induce porosity, as seen in Fig. 1.

Although TPMS structures offer excellent controllability and permeability, the Voronoi tessellation stochastic algorithm possesses a non-uniform, random pore distribution mimicking the design features of trabecular bone [30]. The governing equation supporting the algorithm to produce the stochastic Voronoi tessellation method is shown in Eq. 3 [44], where $\:{P}_{1},\:\dots\:,{P}_{n}$, is the set of distinct seeds in a finite region, $\:{V(P}_{i})$ is the Voronoi polygon associated with $\:{P}_{n}$, and $\:D$ is the Euclidean distance between the points.

3 $$V\left( {{P_i}} \right) = \left\{ \eqalign{& P{\rm{|}}D\left( {P,{P_i}} \right) \le D\left( {P,{P_j}} \right), \cr& \quad i \ne j,andi,j = 1,2,3, \ldots,n \cr} \right\}$$

**Fig. 2:**
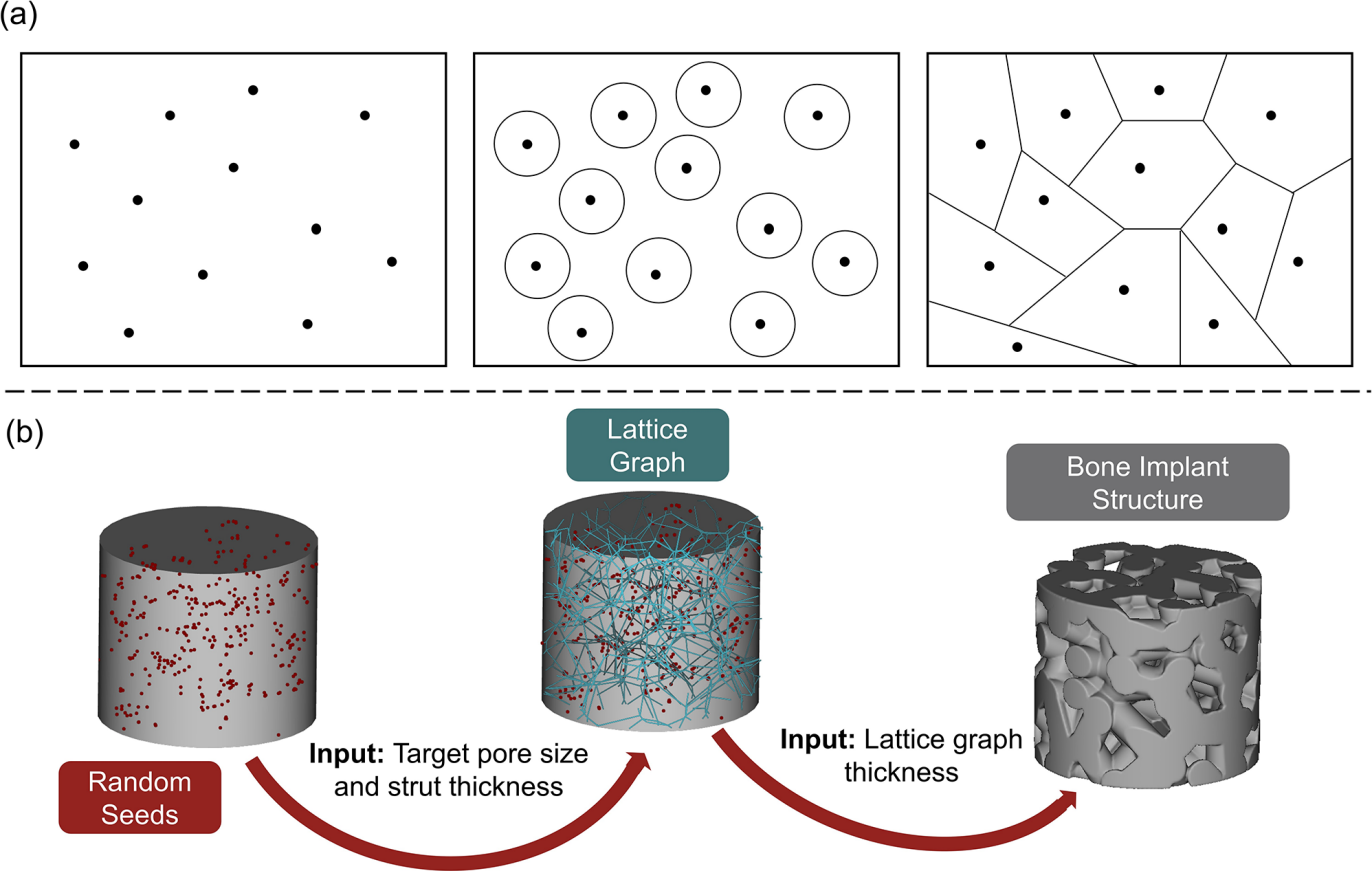
The Voronoi tessellation method design process in 2D and 3D. (**a**) 2D graphical representation of seeds points randomly distributed in a finite space and a radius continuously growing outward until they intersect to form polygons. (**b**) 3D process of the random seeds applied from an input target pore size and beam diameter to create a lattice graph encompassing the random seed points and then thickened to form a bone-like implant structure

The Voronoi tessellation method first randomly distributes seeds in a selected region. The seeds are then encompassed by polygons continuously growing outward until they intersect [29]. The lattice is then thickened to create a 3D structure once applied to a 3D finite space, the design process is shown in Fig. 2.

In all cases, TPMS and Voronoi structures, the chosen porosity was influenced by the dimensions of the cylindrical structure relative to the size of dental implants to maintain the load bearing strength while inducing interconnected channels. Porosity is calculated using Eq. 4, where P is the porosity volume fraction, $\:{V}_{pores}$ is the volume of the porous scaffold structure, and $\:{V}_{solid}$ is the volume of the fully dense structure [45].

4 $$P=\left(1-\frac{{V}_{pores}}{{V}_{solid}}\right)x\:100$$

**Fig. 3:**
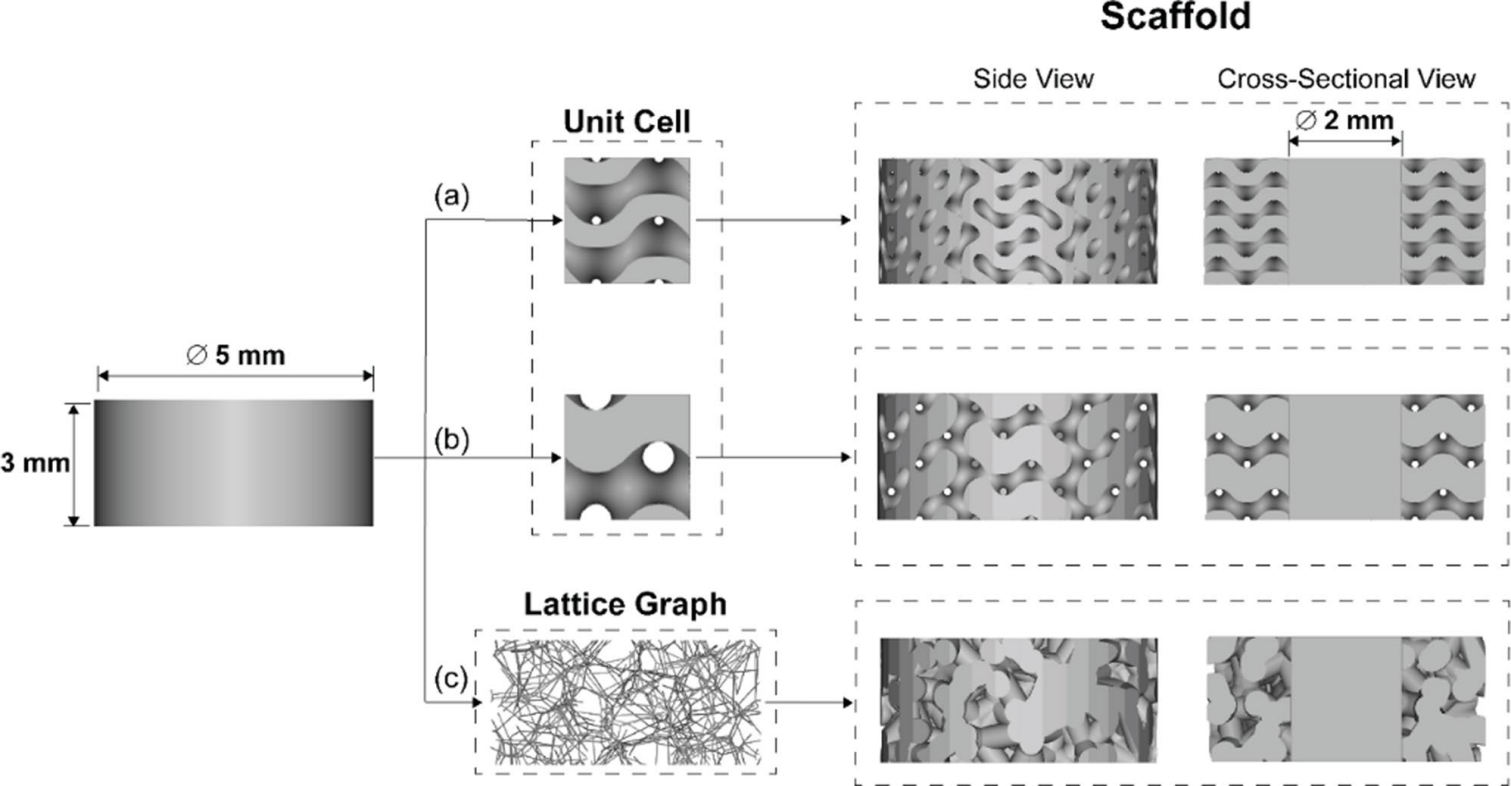
CAD scaffold modeling process of the unit cells and lattice graph of each porous architecture applied to a cylindrical geometry with channels leading to a 2 mm dense core: (**a**) TPMS– Sheet Gyroid, (**b**) TPMS– Solid Gyroid, and (**c**) Voronoi– Stochastic

Figure 3 shows a summary of the three different unit cell lattices applied to the intended geometry for experiments, a 5 mm diameter x 3 mm length cylindrical construct with a 2 mm dense core. The geometry was selected based on the size and geometry of standard dental implants used in clinical practice, ensuring scalability and relevance. Typical dental implants range from 3.5 mm to 5.0 mm in diameter, with abutment diameters around 2.0 mm [46]. The 2.0 mm dense core provides mechanical stability, aligning with the size of the abutment, while the porous outer layer facilitates bone ingrowth. Dental implant size varies depending on the tooth being replaced, and different studies have adopted similar geometries based on the standard sizes of leading manufacturers [47–49]. While the most common length of dental implants ranges from 6 mm to 20 mm [50], a 3.0 mm length was chosen for experimental purposes to fit cell culture dishes, while assessing fabrication resolution [50].

The target pore size, ranging between 200 and 250 μm, aimed to enhance cell proliferation, with a desired porosity level falling within 30–50%. Table 1 summarizes the designs and their target pore size and porosity. Pore size was calculated by the largest sphere size that could fit into the pore without intersecting the wall, and porosity was calculated using Eq. 4. The TPMS sheet network gyroid had a target pore size of 200 μm and 51.10% porosity, the TPMS solid network gyroid had a target pore size of 220 μm and porosity of 32.57%, and the Voronoi stochastic lattice had a target pore size of 250 μm and 38.39% porosity. It is important to acknowledge the challenge of achieving consistent pore size and porosity combinations across all three porous architectures in the software. This challenge stems from the inherent variability in the input parameters of each architecture, which affects the attainment of uniform pore sizes across different designs. For example, TPMS gyroid structures are influenced by parameters such as periodicity (L) and strut length, while variations in solid and sheet components affect the role of wall thickness (c). In Voronoi stochastic structures, input parameters include target pore size and wall thickness, with randomness resulting in an average pore size incorporated into the structure. Given these considerations, a pore size range of approximately 200 μm to 250 μm was proposed. This research aimed to evaluate porous architectures conducive to cellular ingrowth and fabrication controllability using SLM. Therefore, maintaining a pore size within this range was crucial to ensuring an equitable and meaningful comparison of cell interaction and connectivity.

**Table 1:** Target pore size and porosity designed in CAD

Design Type	Target Pore Size (µm)	Target Porosity (%)
TPMS– Sheet Gyroid	200	51.10
TPMS– Solid Gyroid	220	32.57
Stochastic– Voronoi	250	38.39

### Selective laser melting (SLM) process of the lattice structures

**Fig. 4:**
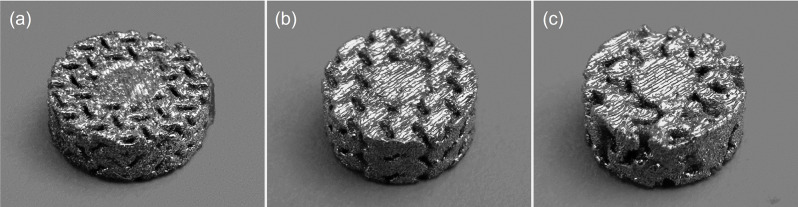
SLM fabricated Ti6Al4V porous samples for in-vitro cell culturing experiments and design validations: (**a**) TPMS sheet gyroid, (**b**) TPMS solid gyroid, and (**c**) Voronoi stochastic

The constructs depicted in Fig. 3 were manufactured using the TruPrint 1000 (Trumpf Inc., Plymouth, USA) SLM machine, with optimized parameters for the Ti6Al4V Grade 23 (AP&C, Quebec, Canada) powder printed in an argon environment. It is important to note that uncontrollable porosity can be generated based on the machine process parameters, namely the laser power, scan speed, and hatch spacing. The selective laser melting parameters influence build part resolution, specifically the scan speed and laser power [51]. High scan speed relative to laser power results in insufficient energy density, leading to unintentional pores termed lack of fusion [52, 53]. Conversely, increasing laser power yields high energy density, forming spherical pores known as keyhole pores, typically smaller than 100 μm in diameter [52]. This research is aimed at fabricating controllable porous structures on the basis of mathematical models using CAD. The optimal machine parameters vary based on spot size and from machine to machine. In our research, we are using SLM as a tool to fabricate microporous scaffolds. Thus, the optimal parameters for producing parts with minimal lack of fusion and keyhole pores with a 99.9% density were obtained from the supplier, Trumpf Inc., of the TruPrint 1000 with 30 μm laser spot size for Ti6Al4V Grade 23. The SLM fabricated TPMS sheet gyroid, TPMS solid gyroid, and Voronoi stochastic structures are shown in Fig. 4. Dimensions for each construct are 5 mm diameter and 3 mm in length with a 2 mm solid core.

### Microcomputed tomography characterization of the fabricated scaffolds

**Fig. 5:**
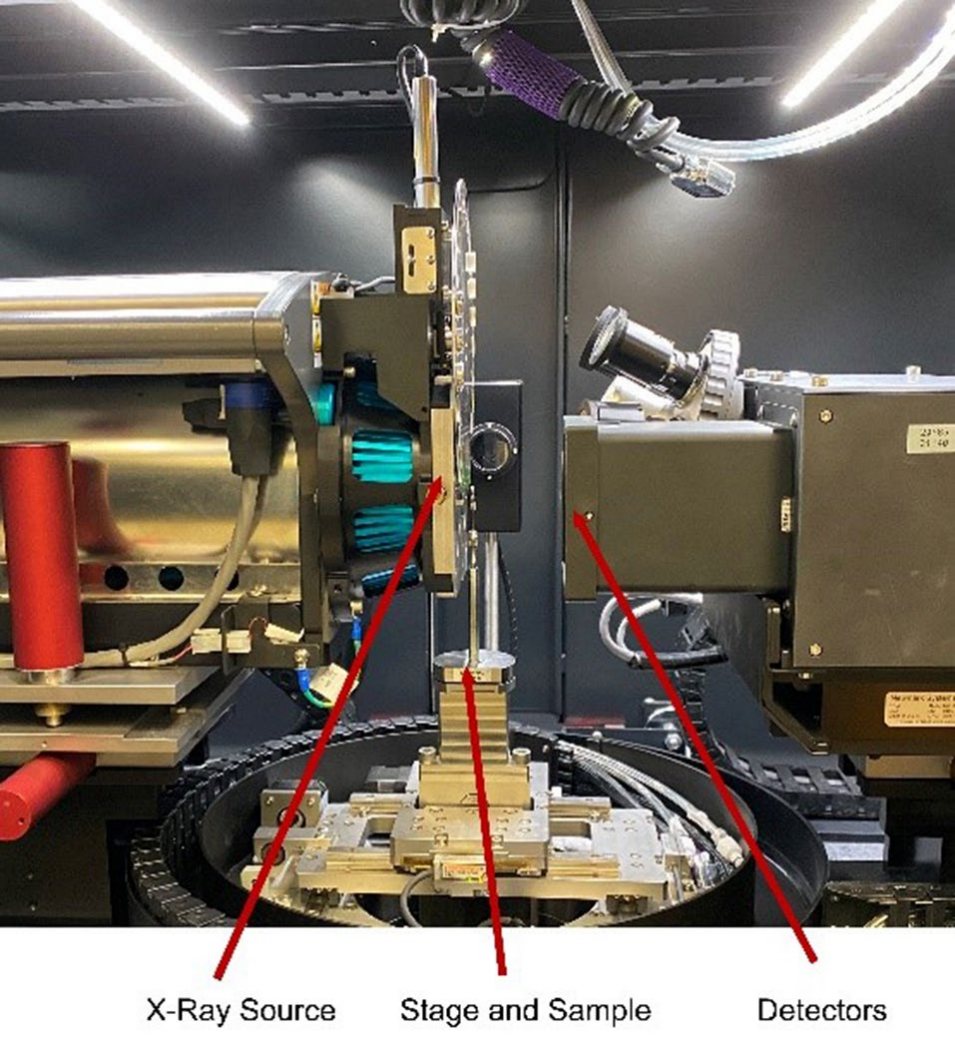
Zeiss XRadia Versa 520 3D X–ray Microscope setup with the samples mounted on a stage between the X-ray source for scanning and detector for magnification adjustments

The quality and resolution of the printed scaffolds are evaluated using microcomputed tomography (microCT) scanning using the Zeiss XRadia 520 Versa (Carl Zeiss X-ray Microscopy Inc., CA, USA) 3D X-ray Microscope, seen in Fig. 5. The TPMS solid and sheet gyroid, and the Voronoi stochastic cylindrical samples, with a diameter of 5 mm and length of 3 mm, were scanned and each data set has a voxel size of 7.49 μm and resolution of 14.98 μm.

Subsequently, the resultant CT scan files were reconstructed using DragonFly 2022.1 (Comet Technologies Canada Inc., Montreal, CA) and Bruker CTan (Bruker, Massachusetts, USA) software, yielding measurements for pore size, porosity volume fraction, pore size distribution, pore connectivity, and 3D models of the geometric structures. The 3D models were inverted in DragonFly to generate solid–modeled pores, enhancing accuracy for analysis in the computational fluid dynamics simulation.

### CFD validation of the connectivity and permeability of the fabricated scaffolds

**Fig. 6:**
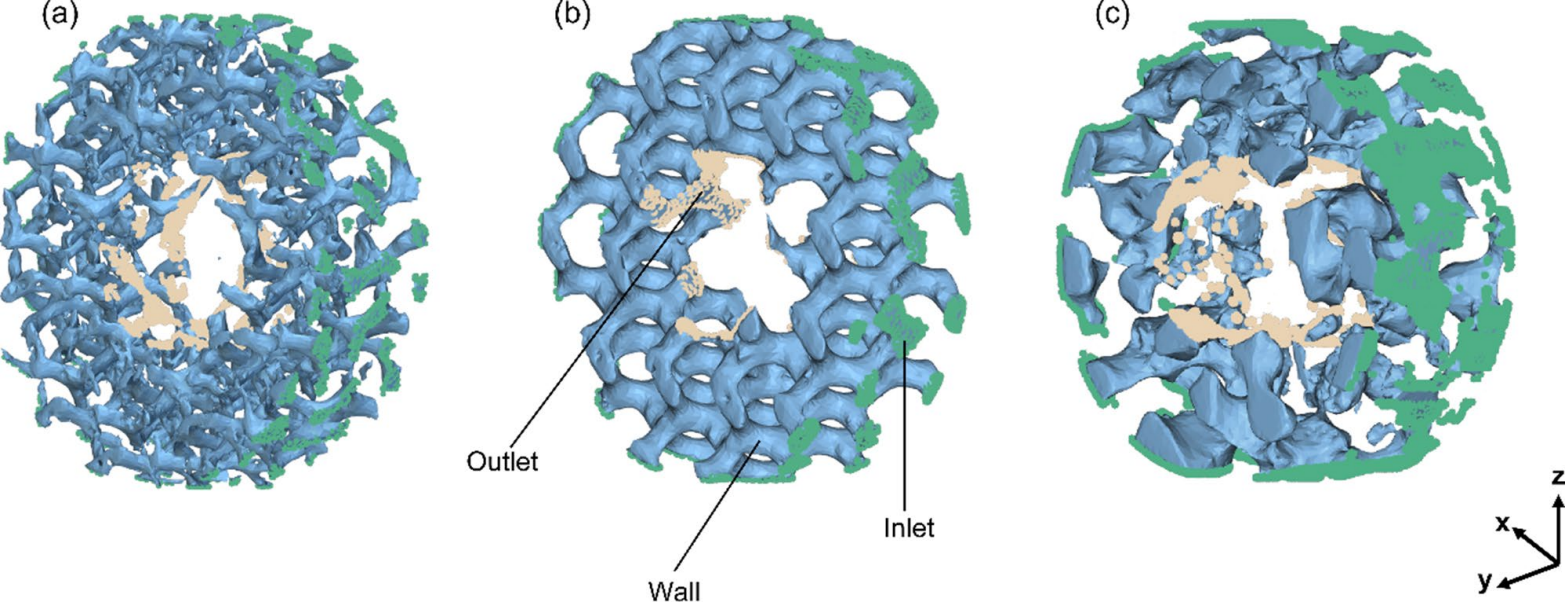
Diagram of the SLM solid–modeled pores from the microCT scans and the boundary conditions: (**a**) TPMS sheet gyroid, (**b**) TPMS solid gyroid, and (**c**) Voronoi stochastic architectures used for the computational fluid dynamics simulation to output permeability. Highlighted in green and light orange nodes are the inlet and outlet, and the solid-modeled pores are taken as the wall

The 3D solid–modeled pores from the microCT scan output of the SLM fabricated scaffolds were evaluated for permeability using Ansys CFX 2024 R2 (Ansys, Inc., Canonsburg, PA, USA) a computational fluid dynamics software. Permeability, defined as the measurement of fluid passage through a porous medium, profoundly influences cell proliferation and vascularization [54]. Figure 6. shows the solid-modeled pores and boundary conditions of the (a) TPMS sheet gyroid, (b) TPMS solid gyroid, and (c) Voronoi stochastic structure.

A single-phase, laminar flow was assumed, and two cases were considered: a static cell seeding environment to mimic the in-vitro cell culturing experiments and a simulation of the environment of the human body. Darcy’s law shown in Eq. 5 is used to calculate permeability where k is the permeability, Q is the volumetric flow rate, µ is the dynamic viscosity, L is the distance of fluid flow through the construct, A is the area of the construct, and ∆P is the pressure gradient from the inlet to outlet [55].

5 $$k = \frac{Q}{A}\frac{{\mu L}}{{\Delta P}}$$

The density and dynamic viscosity of DMEM, 1000 kg/m^3^ and 9.3 × 10^− 4^ kg/m∙s, were used for the case of static cell seeding. The density and dynamic viscosity for whole blood considered at the initial flow during osseointegration, 1050 kg/m^3^ and 4 × 10^− 3^ kg/m∙s was considered for the environment of the human body [56–60]. A constant velocity of 0.01 mm/s was used for static cell seeding conditions, and a constant velocity of 0.7 mm/s was assumed for blood [57, 61] applied at the inlet boundary condition and the outlet pressure was set to 0. The pressure gradient value, ${{\Delta P}}$, was outputted from the ANSYS CFX simulation to calculate the permeability.

### In vitro cell culture

To determine the response of mesenchymal stromal cells to these various morphologies, the 3D printed constructs were seeded with primary bone marrow stromal cells (BMSCs), isolated from 8-week-old wild type C57BL6/J by flushing the bone marrow. Primary cells were cultured in growth media (DMEM, 10% FBS and 1,000 U of penicillin/streptomycin) in tissue-culture-treated polystyrene dishes, grown to confluence, and seeded to constructs at passage 2. Before cell seeding, constructs were sterilized by ethylene oxide gas. Immediately before planting, constructs were soaked in 70% ethanol for 30 min, followed by washing in phosphate-buffered saline (PBS, pH 7.4) and three times in growth media. 300,000 cells were seeded per scaffold in non-treated polystyrene tissue culture dishes (*n* = 4 per group). Cells were allowed to adhere to the Ti disks for 30 min at 37^o^C, then growth media was added to the culture and changed every 48 h. At 72 h, Ti disks were removed from the culture plate into a fresh vessel, and RNA was extracted with 500 uL Trizol reagent according to the manufacturer’s protocol; RNA quantity was assessed by triplicate measurement of A260 using a Beckman spectrophotometer.

Scanning electron microscopy (SEM) and confocal laser microscopy were used to evaluate the distribution and morphology of BMSCs in constructs, at various time points, specimens are fixed in EM-grade paraformaldehyde for 24 h at 4 C.

#### Scanning electron microscopy:

Surface morphology was observed by scanning electron microscopy (JEOL JSM-7800 FLM) with an accelerating voltage of 5 kV and a working distance of 10–15 mm. Before observation, samples were coated with gold using a sputter coater (Desk II, Denton Vacuum Inc.).

#### Confocal laser microscopy:

A Nikon Eclipse C1 microscope is used for all confocal imaging. A 1:1000 solution of Hoescht nuclear stain and 1:200 Alexa-Fluor 488 Phalloidin was applied and incubated for 20 min. Samples were washed three times before imagining them.

## Results

### Manufacturability– Pore characteristics MicroCT scan

**Fig. 7:**
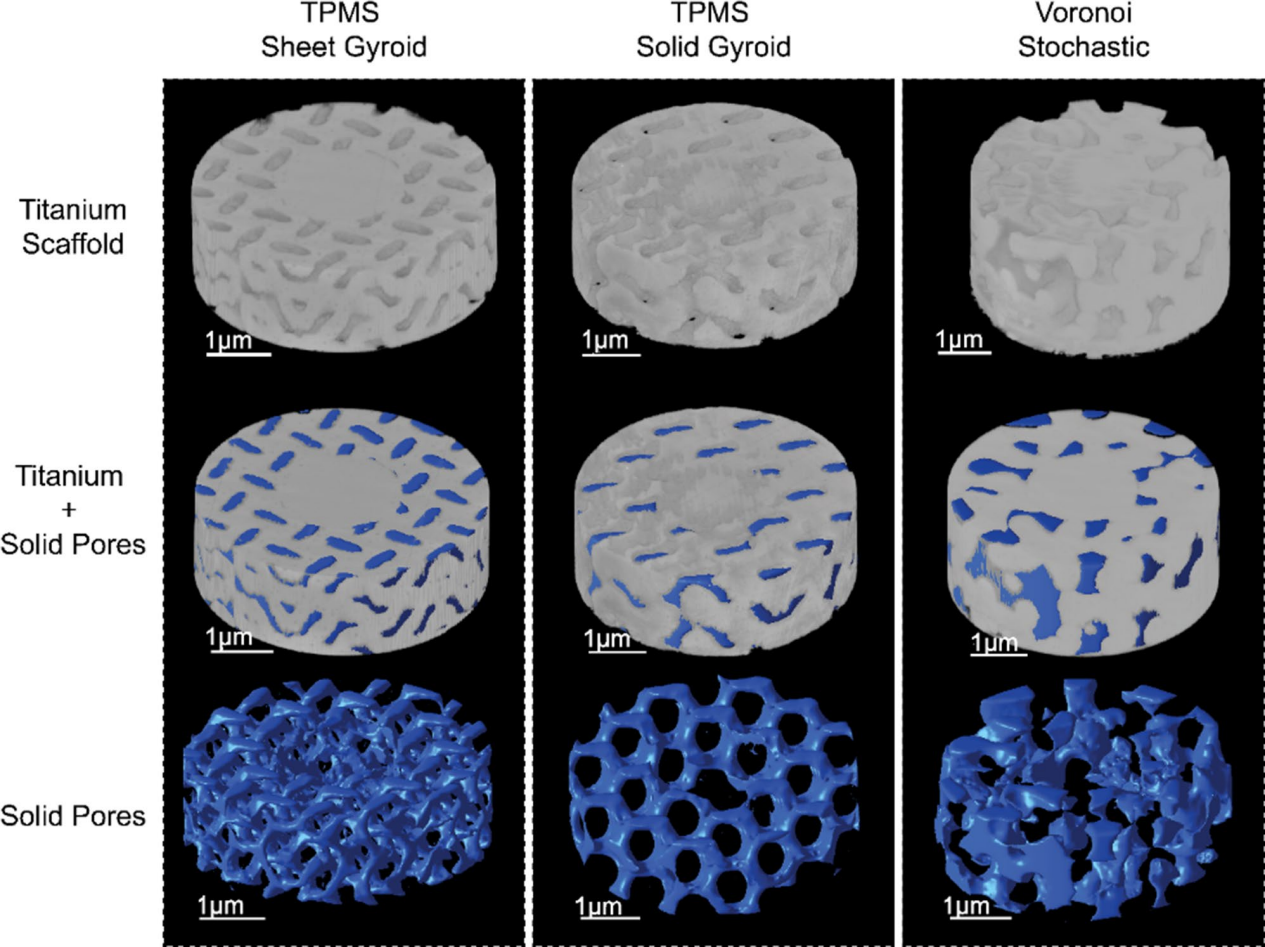
MicroCT scan output models of the Ti6Al4V additively manufactured samples. The solid-TPMS Gyroid, sheet-TPMS Gyroid, and Voronoi stochastic scaffolds modeled in their original form and the voids modeled as a solid

Figure 7 shows the 3D-printed constructs from the microCT scan and the voids modeled as solids to analyze the pore interconnectivity. Qualitatively analyzing the 3D microCT scan of the solid–modeled pores in Fig. 7, the pores are interconnected in both TPMS structures. The stochastic structure is not fully interconnected from the inlet to the core but has the advantage of randomness, like bone.

**Fig. 8:**
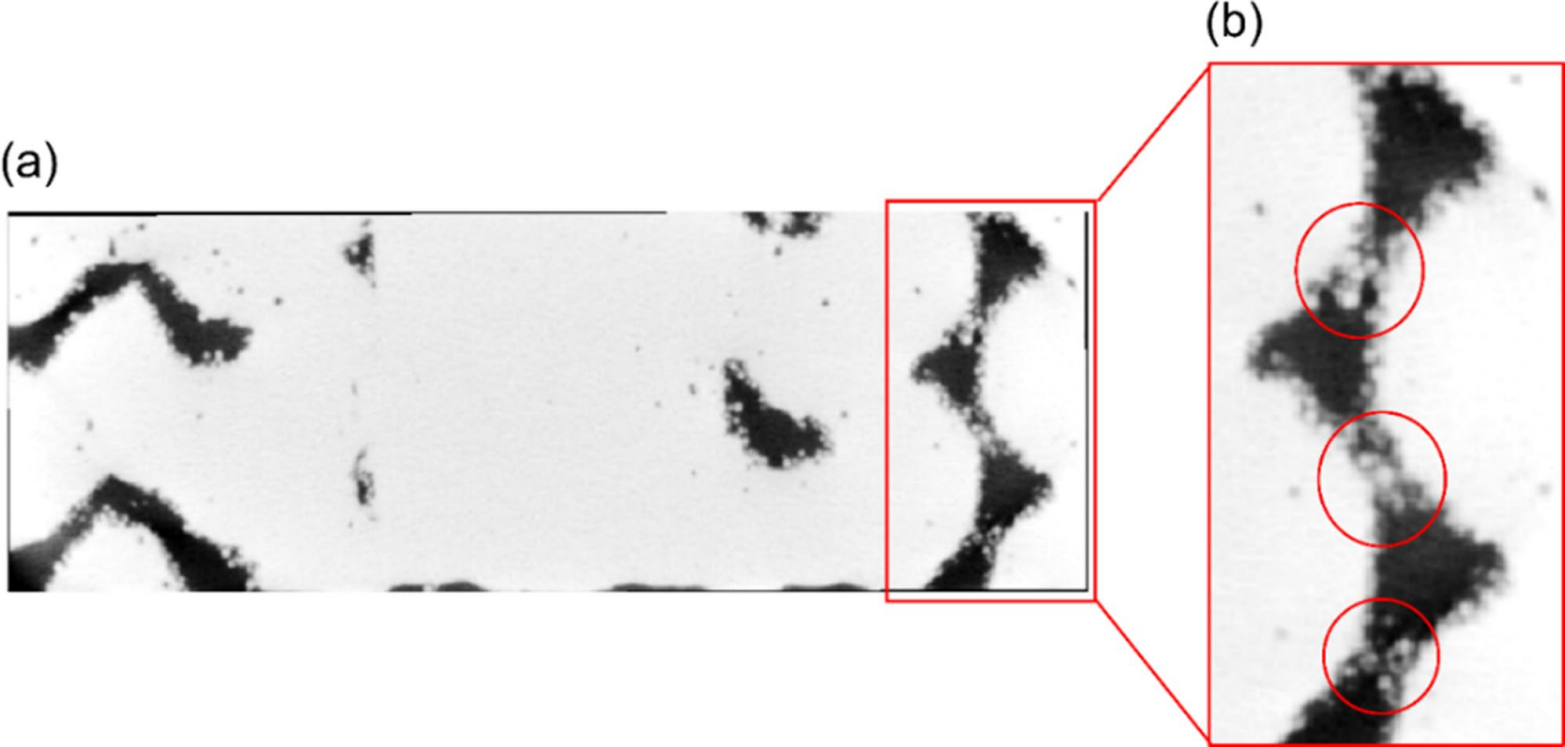
Microcomputed tomography output of the 2D side-view TPMS solid gyroid structure. (**a**) cross-sectional front view, (**b**) enlarged area, a void space from the cross-sectional side view showing the partially melted spherical pores along the walls of the struts

Table 2 shows the manufactured porosity and average pore size for each design analyzed from the models in Fig. 8. In each design, the target pore size fell within the standard deviation, but the porosity decreased from the target by 32.29% for the TPMS sheet gyroid, 12.66% for the solid gyroid, and 19.39% for the Voronoi stochastic structure. The sheet gyroid structure had the largest decrease from the intended porosity attributed to the ability of the printer to produce the complexity of the design. The TPMS solid gyroid was shown to have the highest resolution in terms of pore size and porosity. The precision in the design, fabrication, and overall arrangement of the interconnectivity of the pores is a crucial factor to consider in the unique micro-scale design approach for pores below 300 μm in cylindrical constructs.

**Table 2:** The calculated average pore size and porosity of each manufactured design type

Scaffold	Manufactured Pore Size (µm)	Manufactured Porosity (%)
TPMS– Sheet Gyroid	197.74 ± 91.49	18.81
TPMS– Solid Gyroid	153.28 ± 60.92	19.91
Stochastic– Voronoi	214.90 ± 140.00	19.00

The discrepancy between the intended and actual porosity in Selective Laser Melting (SLM) arises from a specific phenomenon occurring during the process. This phenomenon involves partial melting of the powder particles onto the surface during laser scanning. These partially melted particles adhere to the side and overhang surfaces as adjacent powders are scanned. Despite efforts to optimize process parameters, this effect remains a drawback of SLM [62–64]. We hypothesize that this phenomenon contributes to the discrepancy, resulting in SLM-printed samples being less porous than intended. To evaluate this hypothesis, we conducted microCT scans, and analyzed the 2D slices as depicted in Fig. 8. The scans reveal sphere-shaped partially melted powders adhering to the outer surfaces and within the pores. Consequently, the partially melted powder is considered part of the solid volume fraction, leading to a lower calculated manufactured porosity. This phenomenon is observed consistently across all three structures. Despite not reaching the desired porosity, the intricate porous architectures achieved interconnectivity, resulting in improved biological properties compared to a fully solid structure.

### Porosity distribution

**Fig. 9:**
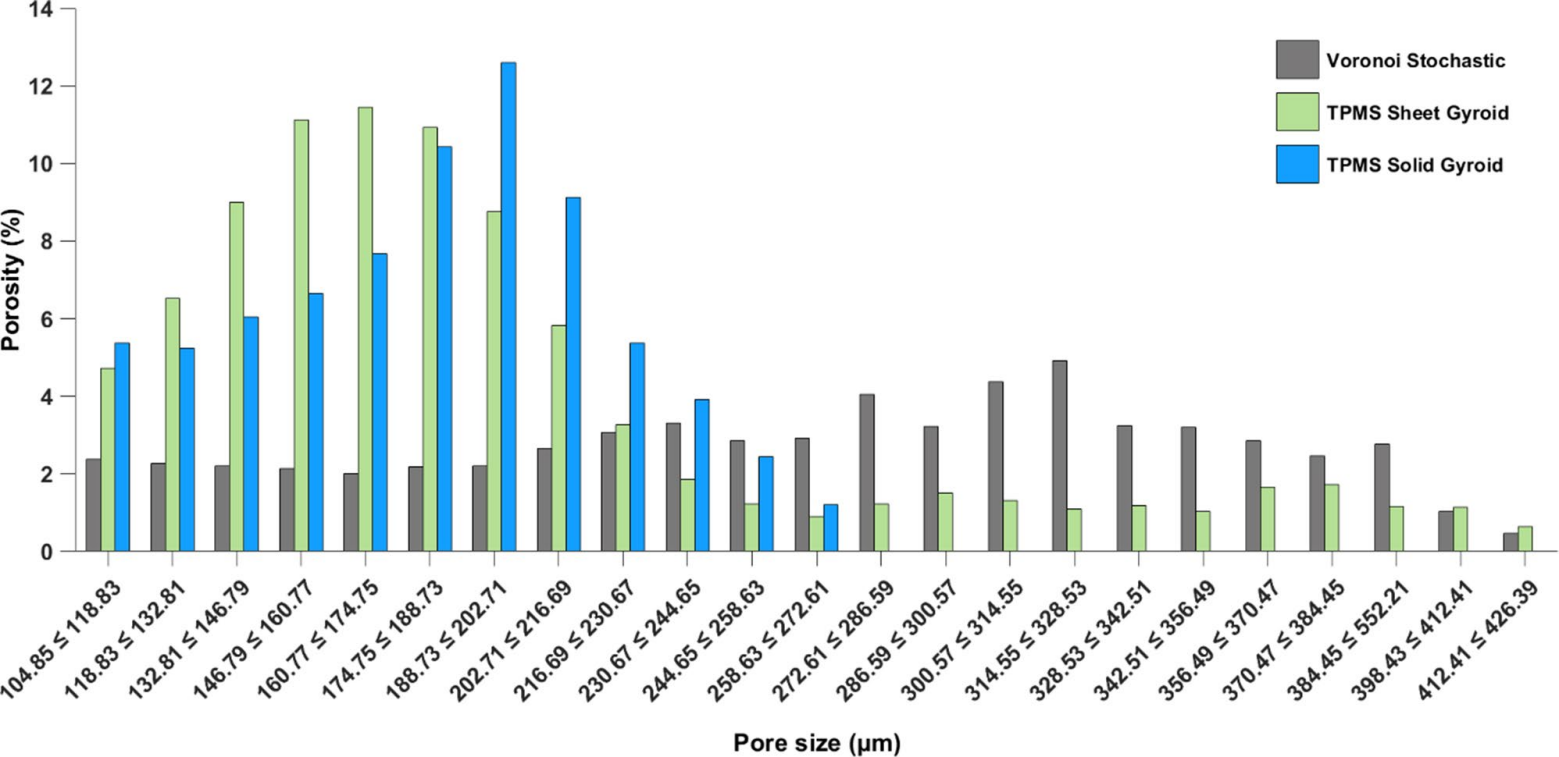
Pore size distribution of the selective laser melted Stochastic Voronoi, TPMS Sheet Gyroid, and TPMS Solid Gyroid structures from microCT scans

**Fig. 10:**
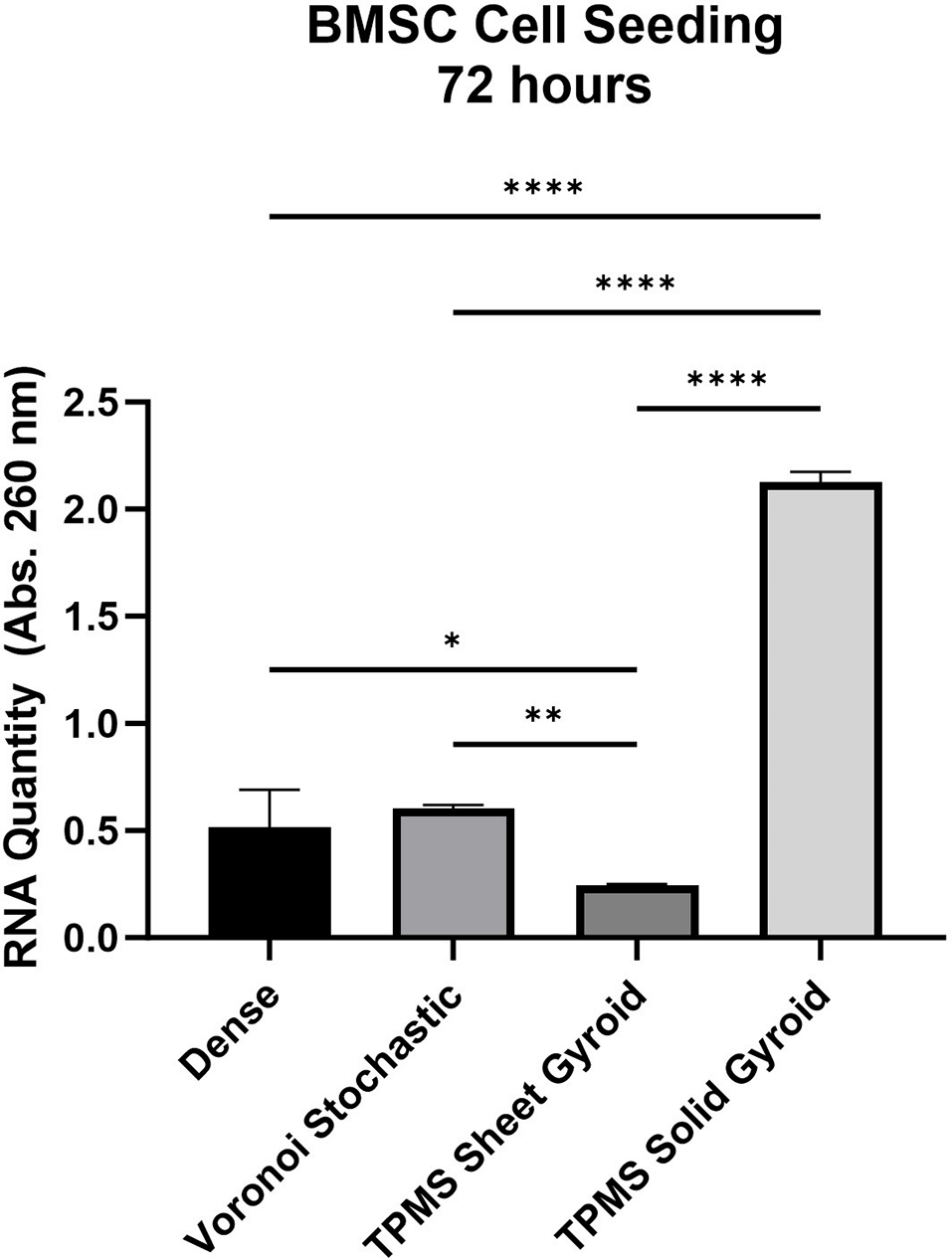
RNA quantity comparison of the dense, Voronoi stochastic, TPMS sheet gyroid, and TPMS solid gyroid at 72 h

The distribution of porosity in complex micro-level designs offers insights into the printing quality and the performance of the printed structures. Figure 10 shows the porosity distribution for each scaffold, data was taken for pores within the range of 100–450 μm because pores with sizes less than 100 μm are attributed to the keyhole and lack of fusion effects during printing and do not significantly affect cell growth [48]. The target pore size for each design falls within the range of 200–250 μm, but the distribution of pore sizes varies for each porous architecture. In Fig. 9, the Voronoi stochastic structure exhibits a random frequency distribution of pores with no specific pore size dominating. The TPMS sheet gyroid shows a distribution centered around approximately 160 μm. The TPMS solid gyroid has the least amount of randomly distributed pores, with the maximum pore size reaching 272.61 μm and a distribution centered around 200 μm.

The TPMS solid gyroid scaffold demonstrates the highest controllability level for designing and manufacturing processes, resulting in greater precision and control over the characteristics and properties of this scaffold design. Despite the intended pore size of 250 μm for the Voronoi stochastic architecture, the resolution of the print is the lowest suggesting that the level of detail and accuracy in reproducing the intended pore size was not as easily achieved.

### In vitro cell culturing validation

The results from in-vitro cell culturing refined the selection for the scaffold based on its ability to promote cell attachment and culture at early time points, mimicking the time soon after dental implant placement where significant cellular proliferation and migration takes place in the wound healing microenvironment. First, it was determined that the TPMS solid gyroid structure has a significantly higher capacity for cell adhesion and maintenance at 72 h based on the amount of extracted RNA (given a standardized cell seeding), as seen in Fig. 10. The absorbance values of the dense (control), Voronoi stochastic, TPMS sheet gyroid, and TPMS solid gyroid are, 0.516 ± 0.175, 0.604 ± 0.015, 0.244 ± 0.008, and 2.153 ± 0.0483, respectively. The TPMS solid gyroid demonstrated a 4-fold increase in its ability to facilitate cell adhesion compared to the fully dense (control), which corresponds to its increased surface area.

**Fig. 11:**
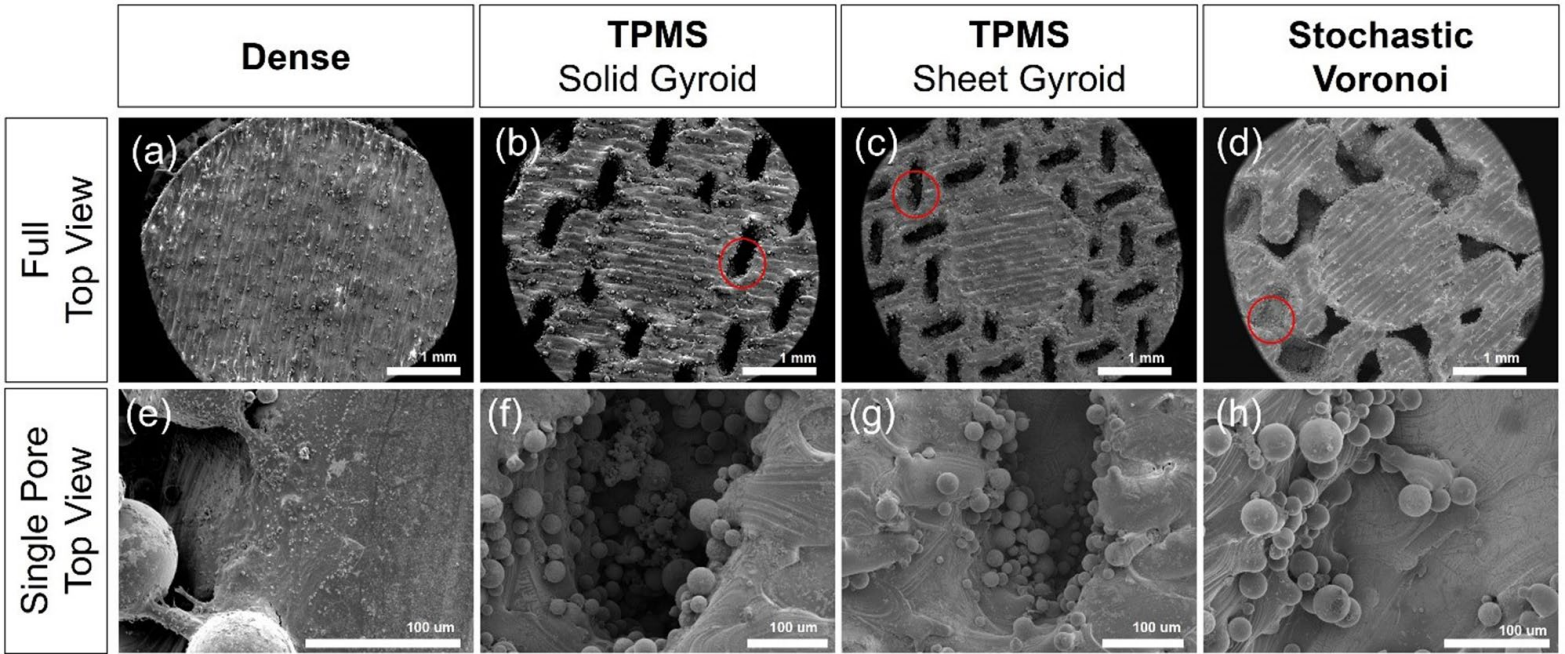
Scanning electron microscopy imaging of the full top view and single pore top view morphology encircled in red. (**a, e**) Dense, (**b, f**) TPMS solid gyroid, (**c, g**) TPMS sheet gyroid, and (**d, h**) Voronoi stochastic scaffolds

To assess the surface morphology, distribution of cells, and adhesion patterns for the dense (control), TPMS solid gyroid, TPMS sheet gyroid, and Voronoi stochastic scanning electron microscopy and confocal microscopy were performed. The results from SEM for each construct are shown in Fig. 11 for a qualitative assessment of the surface morphology and behavior of the cells. These micrographs demonstrate the TPMS solid gyroid has considerably greater pore interconnectivity, which is a critical parameter for cells to colonize the scaffold beyond the porous surface. The increased surface roughness from additive manufacturing increases the material surface favorable for the adhesion of cells. It is noted that the TPMS solid gyroid exhibits a homogeneous distribution of cells throughout the construct in comparison to the TPMS sheet gyroid and Voronoi stochastic architectures, resulting in a basis for a uniform extracellular matrix formation leading to uniform tissue growth, thus explaining the observed increased RNA capacity [65, 66].

**Fig. 12:**
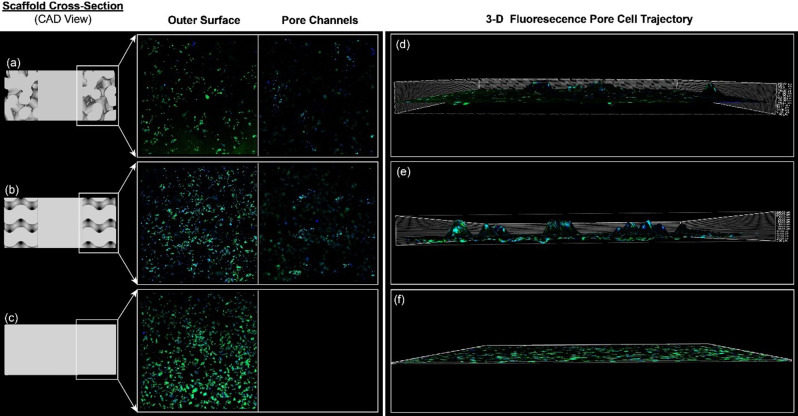
Confocal microscopy of the cell colonization on the SLM printed cross-sections on the outer surface and within the pore channels. The cytoskeleton is stained in green (Aleca Fluor Phalloidin 488) and the nucleus is stained in blue (DAPI). (**a**) Voronoi Stochastic, (**b**) TPMS solid gyroid, and (**c**) dense constructs, (**d**) 3-D fluorescence cell trajectory within a single pore for Voronoi stochastic, (**e**) 3-D fluorescence cell trajectory within a single pore for TPMS solid gyroid, and (**f**) 3-D fluorescence cell trajectory for no porous structure (dense)

**Fig. 13:**
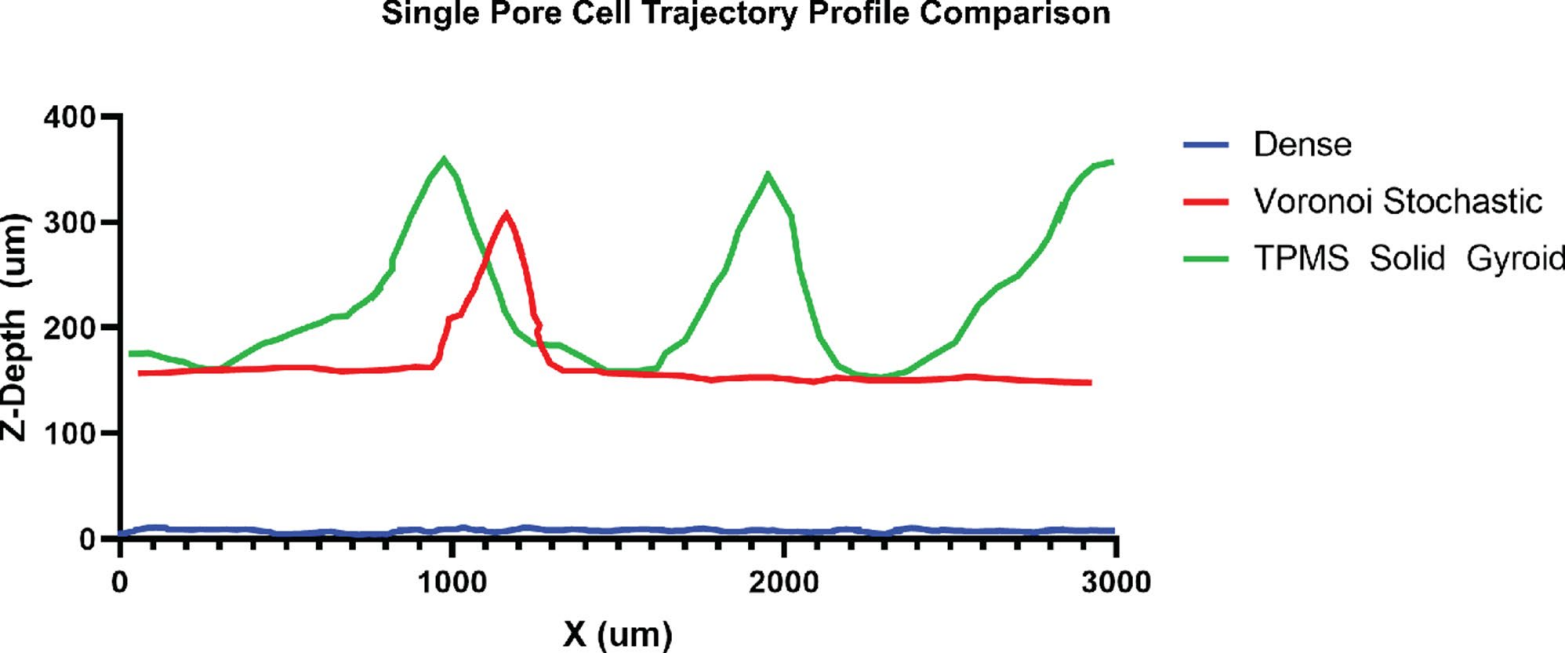
Comparison of the cell trajectory profile within a single pore of the cross-sectional Voronoi stochastic and TPMS solid gyroid scaffolds and on the cross-sectional surface of the dense structure

The TPMS sheet gyroid scaffold was excluded from the fluorescent microscopy experiment and from consideration as a final design based on the suboptimal results of the RNA extraction and SEM observations. The objective was to compare and contrast the non-uniform, random stochastic Voronoi scaffold to the TPMS uniform, periodic scaffold to evaluate their relative advantages. Cross-sections of the TPMS solid gyroid, Voronoi stochastic, and dense structures were printed by SLM to evaluate the pore interconnectivity and cell penetration further, as seen in Fig. 12 (a-c). Confocal laser microscopy assessed the cell distribution profile across the pores. Figure 12 (a) and (b) shows the cell growth across the cross-sections of each porous design and within the pore channels. In Fig. 12(b), the TPMS solid gyroid showed increased cell activity for both the outer surface and pore channels compared to Fig. 12(a), the Voronoi stochastic structure. The Z-profile of the surface of each construct and representative cross sections from the CAD design are shown in Fig. 12(d) and (e). The TPMS solid gyroid and Voronoi stochastic design both illustrate pores capable of facilitating cell growth, as shown by the signal profile along the Z-axis of cell-scaffold constructs. It is relevant to note that a greater number of pores are cellularized in the TPMS gyroid compared to Voronoi stochastic. Additionally, the comparison of the cell trajectory profile within a single pore is shown in Fig. 13. The slope of the Voronoi stochastic is significantly steeper than the TPMS, which may negatively influence cell migration, integration, and occlude vascular ingrowth critical to subsequent bone formation.

### Permeability and streamline interconnectivity

The characteristics of pores play a critical role in determining cell behavior, influencing processes such as cell adhesion, nutrient diffusion, cell proliferation, and bone growth. The solid-modeled pores from the microCT scan of the SLM-printed scaffolds in Fig. 7 were used to comprehensively assess the theoretical behavior of cells using CFD simulation. This study examined the streamline patterns and permeability, coupled with in-vitro cell culturing, to systematically compare permeability and trajectory behavior. Permeability values under static cell seeding and human body conditions were documented in Table 3, using DMEM and human blood as the fluid domain. The TPMS solid gyroid scaffold outperformed the TPMS sheet gyroid and Voronoi stochastic scaffold designs, exhibiting the highest permeability. In this study, the TPMS solid gyroid exhibited a permeability of 9.26 × 10^− 7^ m^2^ and 3.34 × 10^− 7^ m^2,^ as seen in Table 3.

**Table 3:** Permeability output for each porous construct in the environment of cell culturing and human blood

	Input		Permeability (m^2^)		
**Fluid**	**Dynamic Viscosity ** (kg/m·s)	**Inlet Velocity** (mm/s)	**Voronoi** **Stochastic**	**TPMS** Sheet Gyroid	**TPMS ** Solid Gyroid
DMEM	9.30 × 10^− 4^ [56]	0.01 [61]	2.09 × 10^− 10^	1.3 × 10^− 10^	9.26 × 10^− 7^
Human Blood (Bone Marrow Microvessels)	4.00 × 10^− 3^ [57]	0.7 [57]	1.10 × 10^− 10^	1.16 × 10^− 10^	3.34 × 10^− 7^

**Fig. 14:**
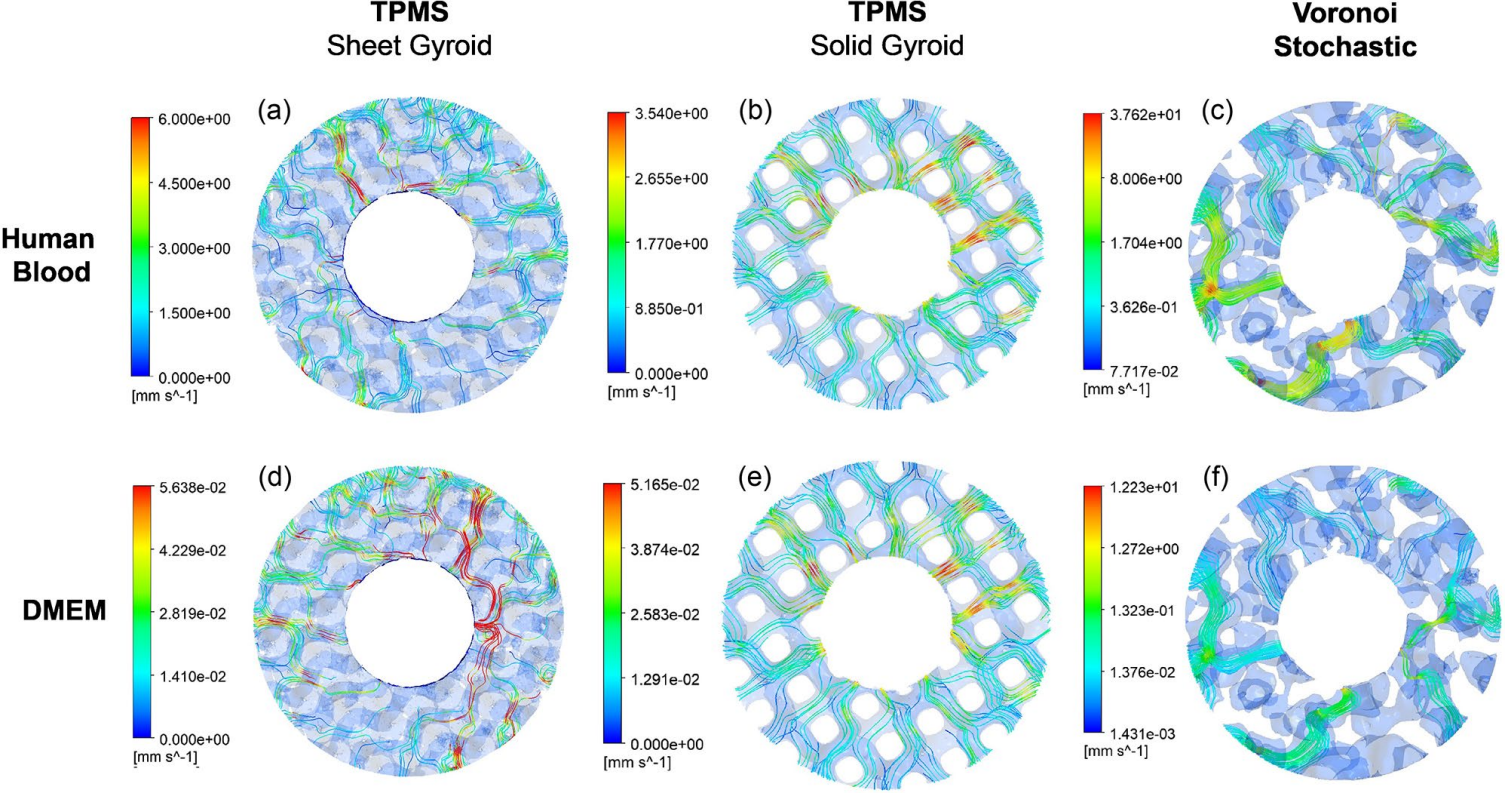
Velocity profile from CFD analysis of the TPMS sheet gyroid, TPMS solid gyroid, and Voronoi stochastic scaffolds: (**a-c**) scaffolds modeled in the environment of human blood; (**d-f**) scaffolds modeled in static cell seeding conditions using DMEM fluid. (**b, e**) The TPMS solid gyroid shows a high volume of flow trajectory from the inlet to the outlet

In addition to permeability, the velocity profiles and trajectory paths of the scaffolds influence the transport of mass and cell attachment [67–71]. Figure 14(a-f) shows the velocity and qualitative trajectory profiles from the computation fluid dynamic simulation of each scaffold in the environments of static cell seeding and the human body. Focusing on the TPMS structures in Fig. 14(a), (b), (d), and (e), the minimal internal fluid rates indicate a more consistent velocity distribution, an attribute conducive to favorable bone growth [70]. The low-velocity profile facilitates robust cell adhesion to the titanium surface, promoting cell clustering and subsequent proliferation. The augmented proliferation positively indicates bone growth, particularly for the initial phases of osseointegration. However, the increasing velocity at the center of the pores shows the acceleration of cell migration to the scaffold core, a crucial factor for deeper bone growth. This phenomenon is consistent across all three porous structures shown in Fig. 14. In Fig. 14(b) and (e), the TPMS solid gyroid demonstrates the highest fluid trajectory with streamline paths at every pore traversing from the inlet to the center.

Overall, the TPMS solid gyroid exhibits a lower internal fluid flow rate and the highest fluid trajectory, suggesting its potential for fostering an environment for enhanced cell growth positioning it as a suitable design for tissue engineering applications. This observation aligns with the results of the in-vitro experiments for the TPMS solid gyroid configuration.

## Discussion

Porous architectures have gained significant attention in tissue engineering due to their ability to enhance cell adhesion, proliferation, and integration. Among the variety of porous designs, the TPMS gyroid and Voronoi stochastic structures have been identified as particularly favorable for orthopedic and dental implant applications [29, 37]. However, the specific architecture of these scaffolds has a profound influence on their biological and mechanical performance, emphasizing the need for comparative evaluation to determine optimal design parameters for manufacturability, design controllability, and cell behavior within the pores. Studies demonstrate that even with similar pore size and porosity, different porous configurations yield varying levels of cell adhesion and proliferation. For instance, Castro et al. and Pires et al. investigated three porous TPMS structure: gyroid, Schwarz diamond, and Schwarz primitive, each designed with 70% porosity. The findings revealed that variations in architecture influenced surface area, and consequently, permeability performance, with each structure demonstrating varying permeabilities [55, 72].

In this study, porous architectures with similar pore sizes and porosity were assessed, demonstrating superior biological interactions within the TPMS solid gyroid. In vitro cell culturing, permeability analysis, and flow trajectory studies indicated that the TPMS solid gyroid promoted increased cell activity, as inferred from the increased RNA quantity observed and the confocal fluorescent cell trajectory results. This aligns with CFD results, where the TPMS solid gyroid exhibited higher permeability values and more consistent fluid flow behavior. These findings suggest that this structure may provide a more favorable environment for bone integration in clinical applications.

Despite the advantages of porous architectures for implant integration, their manufacturability presents practical challenges, particularly in pore resolution and structural complexity. The TPMS solid gyroid structure demonstrated significant advantages due to its highly controllable geometry, enabling precise tuning of pore size. Conversely, the Voronoi stochastic structure, with its randomly distributed pores, exhibited lower resolution and unpredictable spatial distribution, which may limit its clinical practicality. While SLM has been successfully used to manufacture these porous architectures, post-processing steps are often necessary to address residual manufacturing artifacts. For example, acid-etching techniques employed by companies like Zimmer Biomet help reduce bacterial adhesion by modifying surface roughness [73].

Since no standardized porous architecture exists for dental implants, this study provides a comparative framework to evaluate the manufacturability, resolution, design controllability, and cell behavior associated with various architectures. The results establish a foundation for embedding optimized porous architectures into dental implants fabricated using SLM, contributing to a more systematic approach to implant design.

The TPMS solid gyroid exhibited superior performance across all assessed parameters, setting a precedent for a clinically viable dental implant design. By serving as a scaffold for bone regeneration while maintaining load-bearing functionality, the TPMS solid gyroid-based implant could offer a dual-purpose solution for edentulous patients and those with extensive bone defects. Additionally, the ability to tailor pore sizes through additive manufacturing allows for patient-specific customization, further improving clinical outcomes.

This study has several limitations that should be acknowledged. First, the research was limited to in vitro studies, which do not fully replicate the complexities of the in vivo environment. While in vitro experiments provide initial insights into cell viability and scaffold interaction, in vivo experiments give a more comprehensive insight into functional assessments to evaluate bone regeneration and cell differentiation. Future studies should also consider in vivo models with bone loss in medically compromised environments. Another limitation is the lack of direct data on blood viscosity and permeability in the mandible and maxilla. The CFD analysis in this study aimed to assess fluid behavior within the porous constructs, but the assumptions were regarding whole blood viscosity at normal hematocrit levels based on existing literature and textbook values rather than direct measurements from the mandible. Future CFD analysis should consider a medically compromised environment to more accurately reflect blood flow dynamics.

In conclusion, this research compared three porous architectures for a titanium dental implant and identified the TPMS solid gyroid as the optimal design for the potential to enhance biological fixation for implant success in compromised bone environments.

## Conclusion

This research identified the TPMS solid gyroid as the most promising candidate for clinical translation by evaluating the manufacturability, design controllability, resolution, and biological response. Given its improved cell adhesion and activity, permeability, and structural consistency, this architecture has the potential to serve as a dental implant solution for patients with pre-existing diseases negatively affecting alveolar bone integrity.

## Data Availability

The full set of data and the algorithms to obtain them are presented in the PhD Thesis of Rana Dabaja, archived by the Universiyt of Michigan.

## Abbreviations

MicroCT: Microcomputed Tomography
SEM: Scanning Electron Microscopy
SLM: Selective Laser Melting
TPMS: Triply Periodic Minimal Surface
BMSCs: Bone Marrow Stromal Cells
CFD: Computational Fluid Dynamics
ELI: Extra Low Interstitial
